# Evaluation of Bladder Diary Parameters Based on Correlation with the Volume at Strong Desire to Void in Filling Cystometry

**DOI:** 10.1371/journal.pone.0069946

**Published:** 2013-07-29

**Authors:** Sheng-Mou Hsiao, Chin-Fen Hsiao, Chi-Hau Chen, Ting-Chen Chang, Wen-Yih Wu, Ho-Hsiung Lin

**Affiliations:** 1 Department of Obstetrics and Gynecology, Far Eastern Memorial Hospital, Banqiao, New Taipei, Taiwan; 2 Department of International Business Administration, University of Kang Ning, Tainan, Taiwan; 3 Department of Obstetrics and Gynecology, National Taiwan University College of Medicine and National Taiwan University Hospital, Taipei, Taiwan; Northwestern University, United States of America

## Abstract

Accurate assessment of patient bladder capacity is important in determining the proper initial treatment for lower urinary tract dysfunctions and as well as for monitoring therapeutic outcomes. However, urodynamic study is an invasive procedure. Thus, it is important to find a surrogate for invasive urodynamic study, and the aim of this study is to identify the parameter from patient-recorded bladder diary that is best correlated to the volume at strong desire to void (VSD) derived from urodynamic studies. A total of 900 women who underwent urodynamic studies at a university hospital between January 2009 and December 2011. Correlation between bladder diary parameters and VSD was investigated by Spearman rank-correlation coefficient. Days 1 to 3 average maximum daytime voided volumes excluding the first morning void (DVVmaxavg) (mean 263 ml) had the highest correlation with VSD (mean 261 ml; ρ = 0.51, p<0.001). The predictive value of VSD was 146+0.44 × DVVmaxavg. The days 1, 2, and 3 daytime maximum voided volumes excluding the first morning void (DVVmax) were all significantly associated with VSD and had similar mean volumes (ρ = 0.43–0.46, all p<0.001). DVVmaxavg had the highest area under the receiver operating characteristic curve (0.75; 95% confidence interval = 0.72–0.78) for predicting bladder oversensitivity. The threshold of DVVmaxavg <250 ml had good predictive value for detecting bladder oversensitivity (sensitivity 70.9%; specificity 65.8%), and day 1 DVVmax <250 ml had similar sensitivity (70.6%) and specificity (59.1%). Besides, the correlation coefficients (ρ) between day 1, day 2 and day 3 DVVmax and DVVmaxavg were good with a range of 0.70–0.89. In conclusion, DVVmaxavg was the bladder diary parameter best correlated with VSD. DVVmaxavg and day 1 DVVmax may be useful in screening for bladder oversensitivity.

## Introduction

The pathophysiology of lower urinary tract dysfunction (LUTD) can involve either bladder or urethral problems, and distinguishing these subgroups of female LUTD is essential for providing proper treatment. Unfortunately, some kinds of LUTD can produce similar clinical symptoms. For example, overactive bladder syndrome and polyuria can have increased urinary frequency as a symptom. Symptom-based treatment can be ineffective and lead to drug-related adverse side effects, such as dry mouth, pruritus, and constipation [1]. Accurate assessment of patient bladder capacity may be important in determining the proper initial treatment for LUTD and as well as for monitoring therapeutic outcomes [2], [3]. Bladder capacity can be determined from a bladder diary, by uroflowmetry, or by filling cystometry [4].

Cystometric maximum bladder capacity is a frequently used metric for evaluating therapeutic effects [5]–[9]. However, most people go to the toilet when they have a strong desire to void and do not wait until a sensation of urgency. In addition, we have reported that in women with stress incontinence, infusion of a strong-desire-water-volume in a 20-minute pad test produced better sensitivity than that of infusion of 250 ml water [10], [11]. Thus, the volume at strong desire to void (VSD) determined from filling cystometry is an important clinical parameter. However, many primary care physicians do not have the equipment for performing uroflowmetry or urodynamic studies. Therefore, the aim of this study was to identify the bladder diary parameters that are best correlated to VSD.

## Materials and Methods

This study was approved by the National Taiwan University Hospital Research Ethics Committee. Informed consents of our participants were not obtained due to that the data were analyzed anonymously and this is a retrospective study, and the National Taiwan University Hospital Research Ethics Committee waived the need for written informed consent form the participants. In general, we excluded women of less than 18 year-old, active urinary tract infections, proven bladder cancer or stones, or neurogenic lesions (such as spinal cord injury or stroke) to undergo urodynamic studies in our department. Women who underwent urodynamic studies between January 2009 and December 2011 at a university hospital were retrospectively included in this study. We reviewed their medical records, including clinical data, results of urodynamic studies, and 3-day bladder diaries. If the patients had received treatment for their lower urinary tract symptoms, post-treatment urodynamic studies and bladder diaries were also reviewed.

Bladder diary data included voided volume, time of voiding, and time to sleep. Maximum voided volume was referred to be the largest voided volume during 24 hour (i.e., including daytime and nighttime) bladder diary. It has been previously reported that the largest voided volumes during the day tend to occur in the morning because there is a decreased awareness of bladder filling during sleep. Therefore, the bladder capacity in the morning differs from the “normal” bladder capacity [4], [12]. Because of this, we excluded the first morning voided volume in determining the maximum daytime voided volume, and DVVmax is referred to be the largest voided volume during the daytime voided volumes excluding the first morning void. DVVmaxavg means the average voided volume of day 1, 2, and 3 DVVmax. In addition, VVmax is referred to be the largest voided volume during the 24-hour voided volumes, and VVmaxavg means the average voided volume of day 1, 2, and 3 VVmax in this study.

We performed urodynamic studies with the women in a seated position using a Life-Tech six-channel monitor with computer analysis and the Urolab/Urovision System V (Houston, Texas, USA). These studies included uroflowmetry, filling cystometry (at a rate of 60 ml/min), voiding cystometry (with an infusion of 35°C distilled water), and stress urethral pressure profiles (with a strong-desire volume of distilled water in the bladder). All terminology used in this paper conforms to the standards recommended by the International Urogynecological Association and International Continence Society joint report [13]. All procedures were performed by an experienced technician, and the data were interpreted by a single observer to avoid inter-observer variability. Bladder oversensitivity was defined as an early strong desire to void, corresponding to VSD <250 ml during filling cystometry [13], [14].

STATA software (Version 11.0; Stata Corporation, College Station, Texas, USA) was used for statistical analysis. Wilcoxon signed-rank test and Spearman rank-correlation coefficient were used, where appropriate. A p value <0.05 was considered statistically significant. ROC curve analysis was performed to identify the optimal maximum voided volume of bladder diaries for detecting bladder oversensitivity.

## Results

We reviewed urodynamic and bladder diary data from a total of 900 women, and clinical diagnoses or indications included overactive bladder syndrome (n = 381), overactive bladder syndrome and stress urinary incontinence (n = 297), stress urinary incontinence (n = 175), voiding dysfunction (n = 35) and miscellaneous indications (such as evaluation for occult incontinence in women with pelvic organ prolapse, n = 12, Table 1).

**Table 1:** Comparisons of baseline, cystometric and bladder dairy parameters for women underwent urodynamic studies (n = 900).

Diagnoses or indications	OAB	OAB+SUI	SUI	VD	Miscellaneous	*P* †
Number of patients	381	297	175	35	12	–
Age (years)	56.9±13.4	58.5±11.6	58.3±12.5	57.5±14.5	56.6±13.7	0.55
Parity	3.0±1.4	3.1±1.6	3.2±1.6	3.1±1.1	3.1±1.6	0.74
FD (ml)	135±49	146±50	162±45	156±54	171±39	<0.001
ND (ml)	179±61	192±64	219±63	206±71	233±48	<0.001
VSD (ml)	238±86	263±98	299±99	277±95	321±88	<0.001
Urgency (ml)	304±103	320±107	396±114	350±108	406±96	<0.001
Day 1 VVmax (ml)	300±132	326±153	387±167	309±129	320±144	<0.001
Day 2 VVmax (ml)	306±134	328±151	405±178	299±107	299±128	<0.001
Day 3 VVmax (ml)	307±142	329±156	403±186	328±148	357±149	<0.001
The maximum of days1–3 VVmax (ml)	356±153	388±168	472±193	388±129	383±151	<0.001
VVmaxavg (ml)	304±122	328±135	398±156	312±105	325±129	<0.001
Day 1 DVVmax (ml)	244±107	268±127	317±136	271±109	272±148	<0.001
Day 2 DVVmax (ml)	244±107	252±119	312±136	270±93	254±126	<0.001
Day 3 DVVmax (ml)	236±109	257±124	311±147	294±142	289±140	<0.001
The maximum of days1–3 DVVmax (ml)	287±118	312±140	381±152	356±126	324±157	<0.001
DVVmaxavg (ml)	242±95	259±107	313±119	278±89	271±126	<0.001

† ANOVA with Bonferroni correction: all cystometric and bladder diary parameters - OAB vs. SUI, *P*<0.001; OAB +SUI vs. SUI, *P*<0.001; the other diagnosis/indications subgroup pairs are not uniformly significant.

Values are given as mean ± standard deviation. DVVmax = maximum daytime voided volume excluding the morning void; DVVmaxavg = the average of day 1 to day 3 DVVmax; FD = the volume at first desire to void; ND = the volume at normal desire to void; OAB = overactive bladder syndrome; SUI = stress urinary incontinence; VD = voiding dysfunction; VSD = the volume at strong desire to void; VVmax = maximum voided volume; VVmaxavg = the average of day 1 to day 3 VVmax.

**Figure 1:**
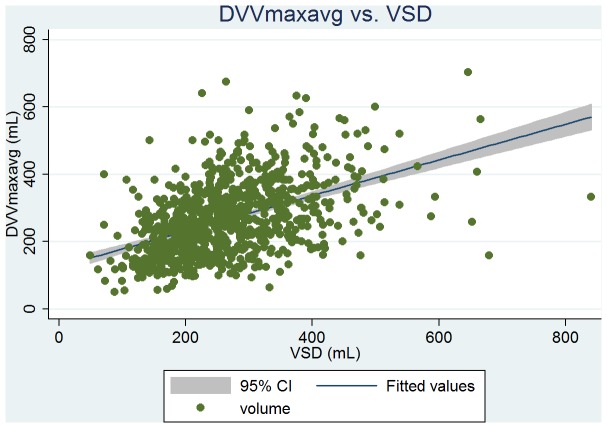
Regression fit scatter plot of the average of day 1 to day 3 daytime maximum voided volumes excluding the first morning void (DVVmaxavg) vs. the volume at strong desire to void (VSD).

**Figure 2:**
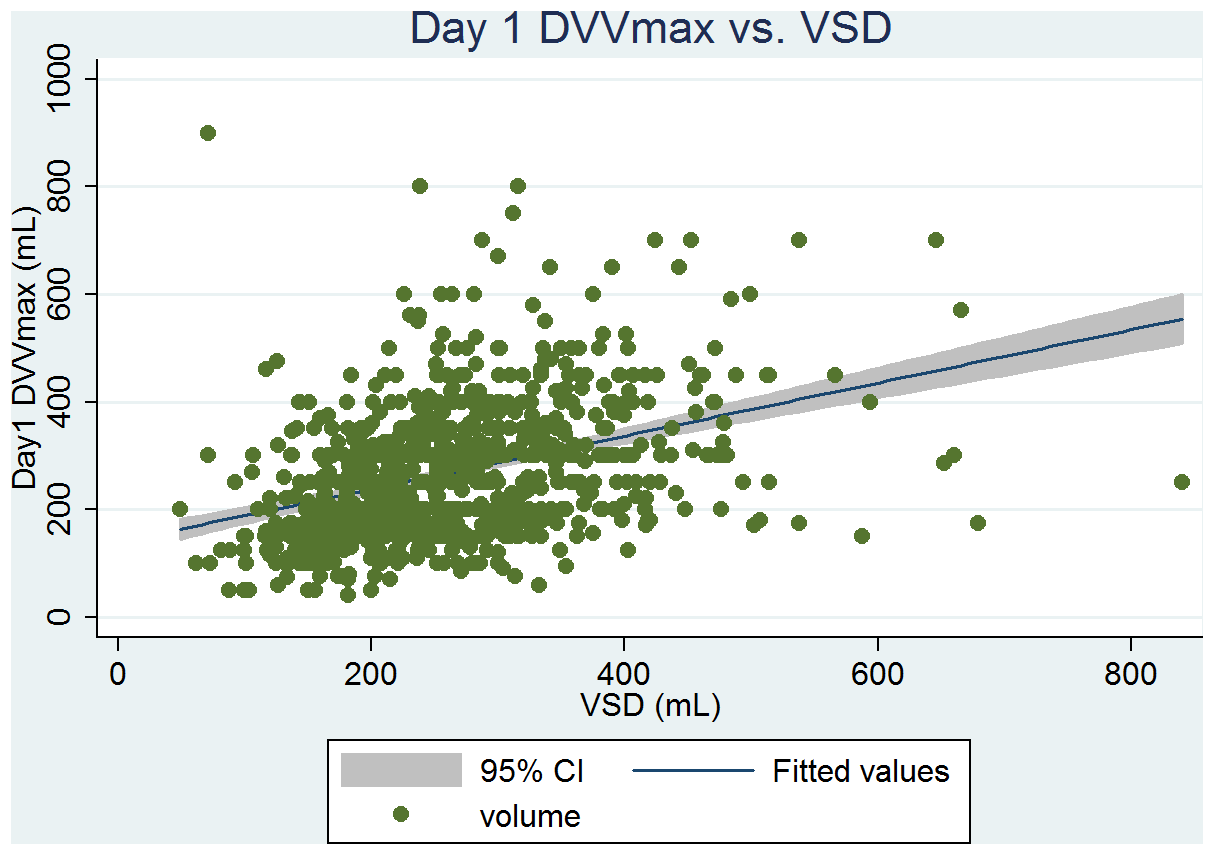
Regression fit scatter plot of day 1 daytime maximum voided volume excluding the first morning void (day 1 DVVmax) vs. VSD.

**Figure 3:**
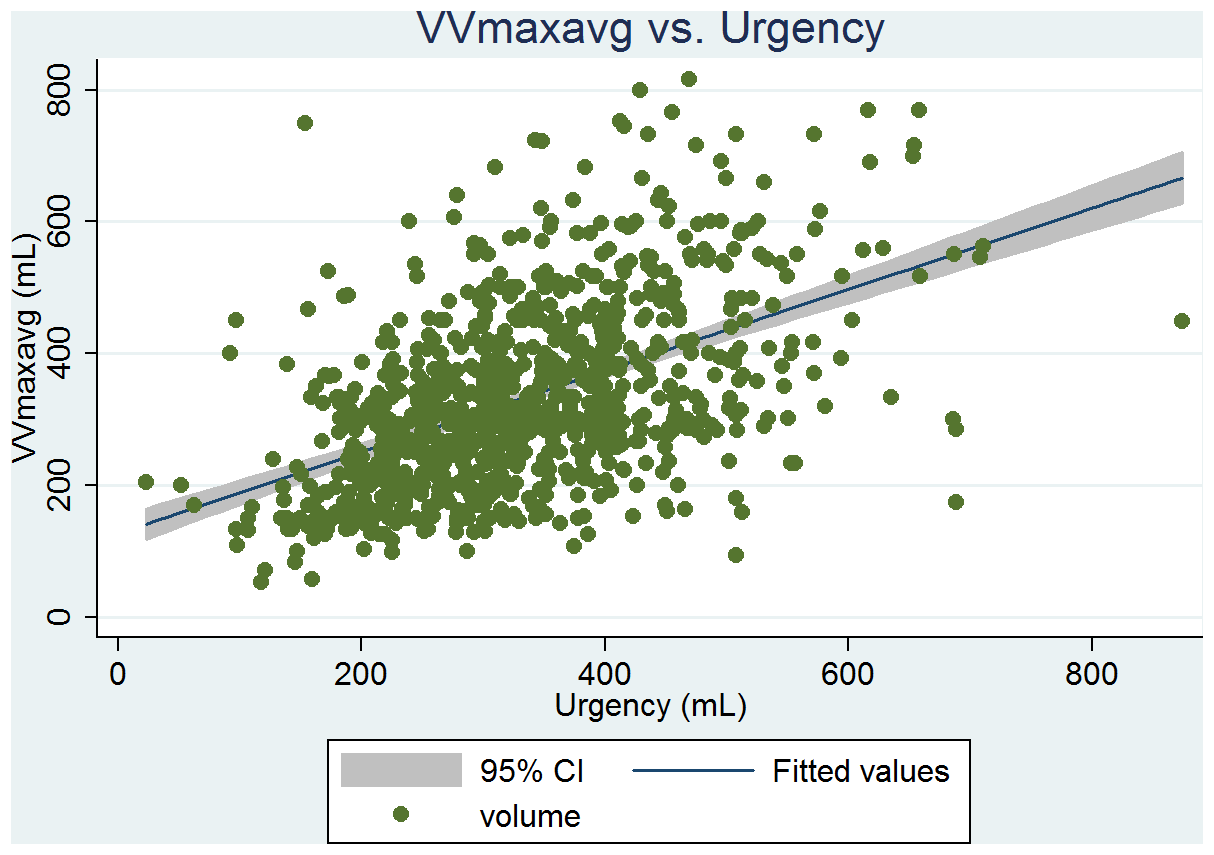
Regression fit scatter plot of the average of day 1 to day 3 maximum voided volumes (VVmaxavg) vs. the volume at urgency.

**Figure 4:**
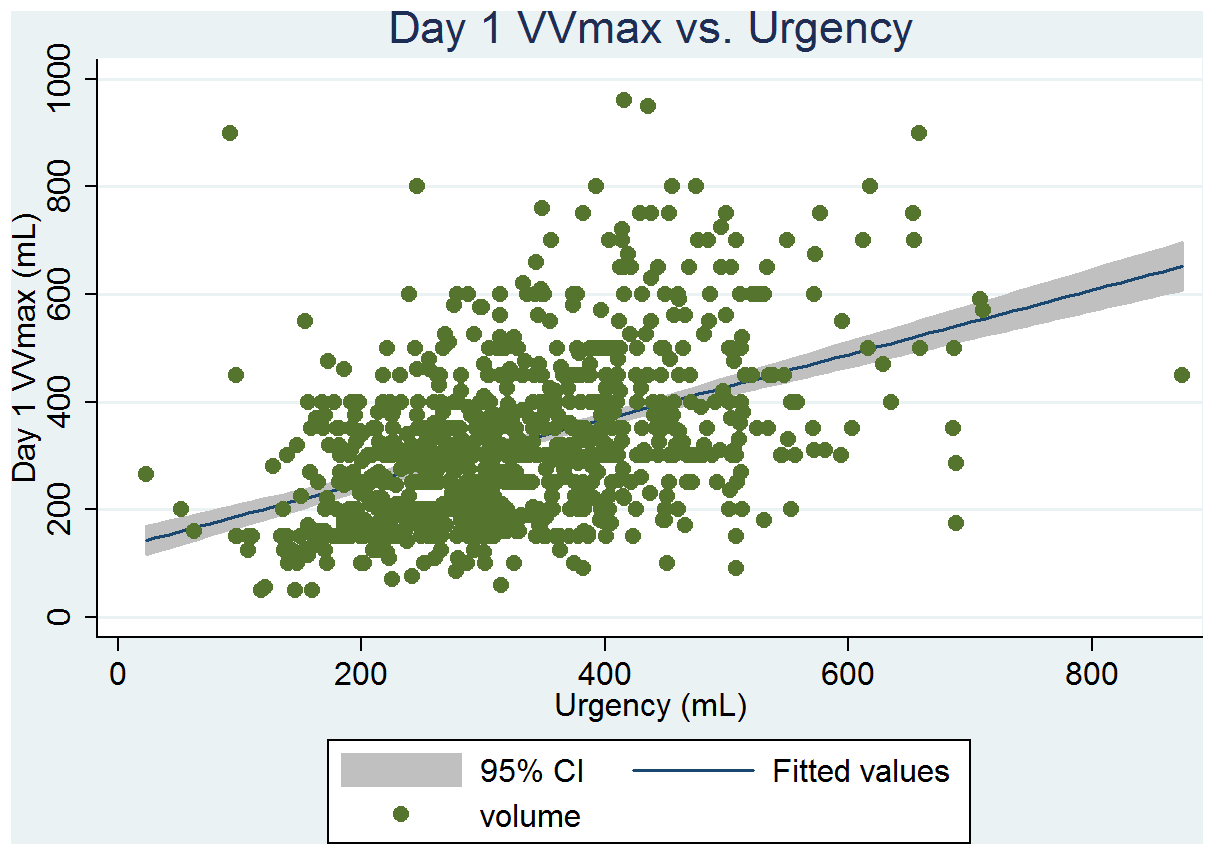
Regression fit scatter plot of day 1 maximum voided volume (VVmax) vs. the volume at urgency.

Baseline clinical, urodynamic, and bladder diary variables are tabulated in Table 2. DVVmaxavg had the similar mean values and highest correlation with VSD at ρ = 0.51 (263 vs. 261 mL); and VVmaxavg had the similar mean values and high correlation with the volume at urgency (331 vs. 330 mL, ρ = 0.50). From the linear regression analysis, we found that the predictive value of VSD was 146+0.44 × DVVmaxavg (P<0.001, Figure 1), and the predictive value of the volume at urgency was 194+0.41 × VVmaxavg (P<0.001, Figure 2). DVVmax for days 1, 2, and 3 were all significantly associated with VSD with similar mean volumes. The predictive value of VSD was 179+0.31 × day 1 DVVmax (P<0.001, Figure 3), and the predictive value of the volume at urgency was 221+0.34 × day 1 VVmax (P<0.001, Figure 4).

**Table 2:** Baseline clinical, urodynamic, and bladder diary variables and correlations between the volume at strong desire to void (VSD) and bladder diary variables in 900 women with lower urinary tract symptoms.

Variables	Values	ρ† for FD	ρ† for ND	ρ† for VSD	ρ† for Urgency
Age (years)	57.7±12.7	–	–	–	–
Parity	3.1±1.5	–	–	–	–
FD (ml)	145±49	–	–	–	–
ND (ml)	193±64	–	–	–	–
VSD (ml)	261±96	–	–	–	–
Urgency (ml)	330±112	–	–	–	–
Day 1 VVmax (ml)	326±150	0.37	0.40	0.42	0.45
Day 2 VVmax (ml)	332±152	0.39	0.41	0.43	0.45
Day 3 VVmax (ml)	334±160	0.42	0.43	0.47	0.48
The maximum of days 1–3 VVmax (ml)	391±170	0.41	0.43	0.47	0.48
VVmaxavg (ml)	331±137	0.43	0.45	0.49	0.50
Day 1 DVVmax (ml)	268±122	0.38	0.42	0.43	0.45
Day 2 DVVmax (ml)	261±119	0.41	0.43	0.46	0.48
Day 3 DVVmax (ml)	260±126	0.43	0.44	0.46	0.45
The maximum of days 1–3 DVVmax (ml)	317±137	0.41	0.44	0.47	0.49
DVVmaxavg (ml)	263±107	0.46	0.48	0.51	0.52

† Each cystometric and bladder dairy parameters are compared with Spearman rank-correlation coefficient, and all *P*<0.001.

Values are given as mean ± standard deviation or correlation coefficient.

Abbreviations same as Table 1.

**Figure 5:**
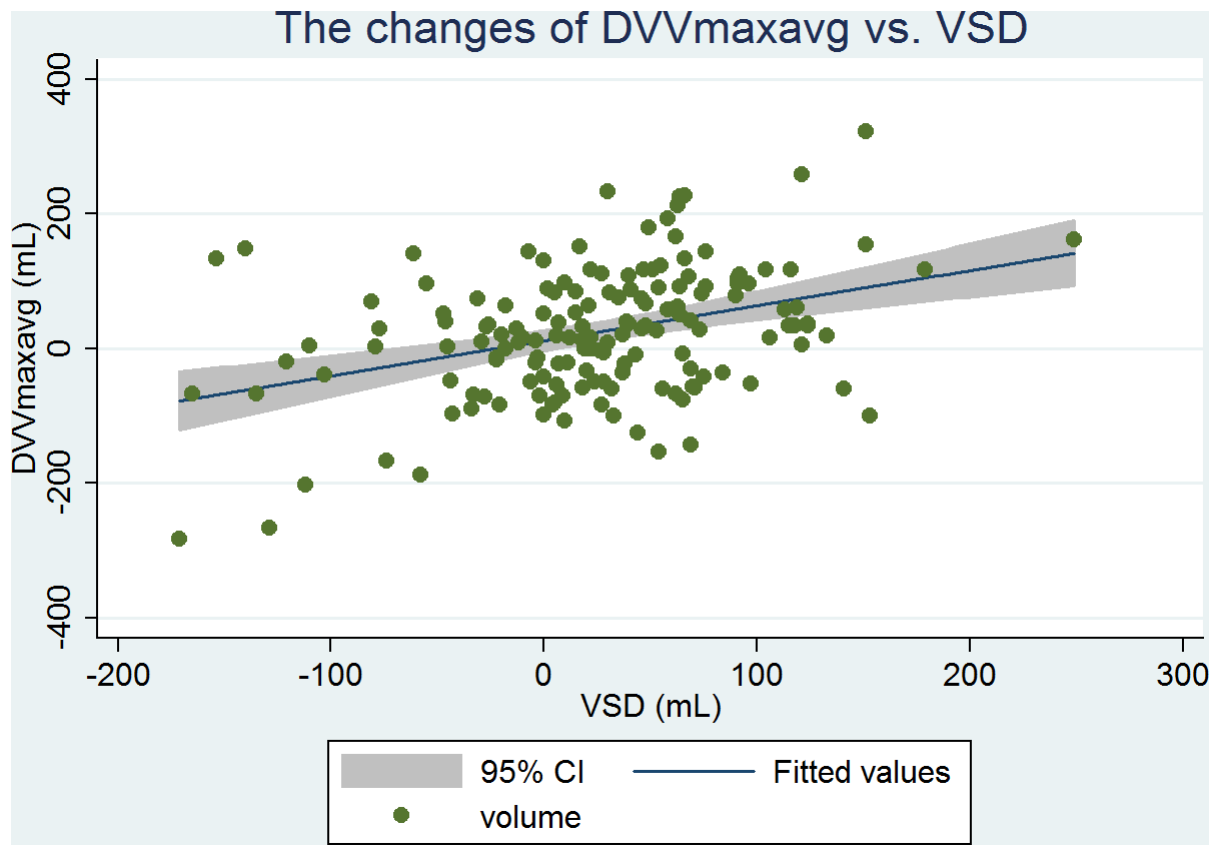
Regression fit scatter plot of the change from baseline in DVVmaxavg vs. the change from baseline in VSD.

**Figure 6:**
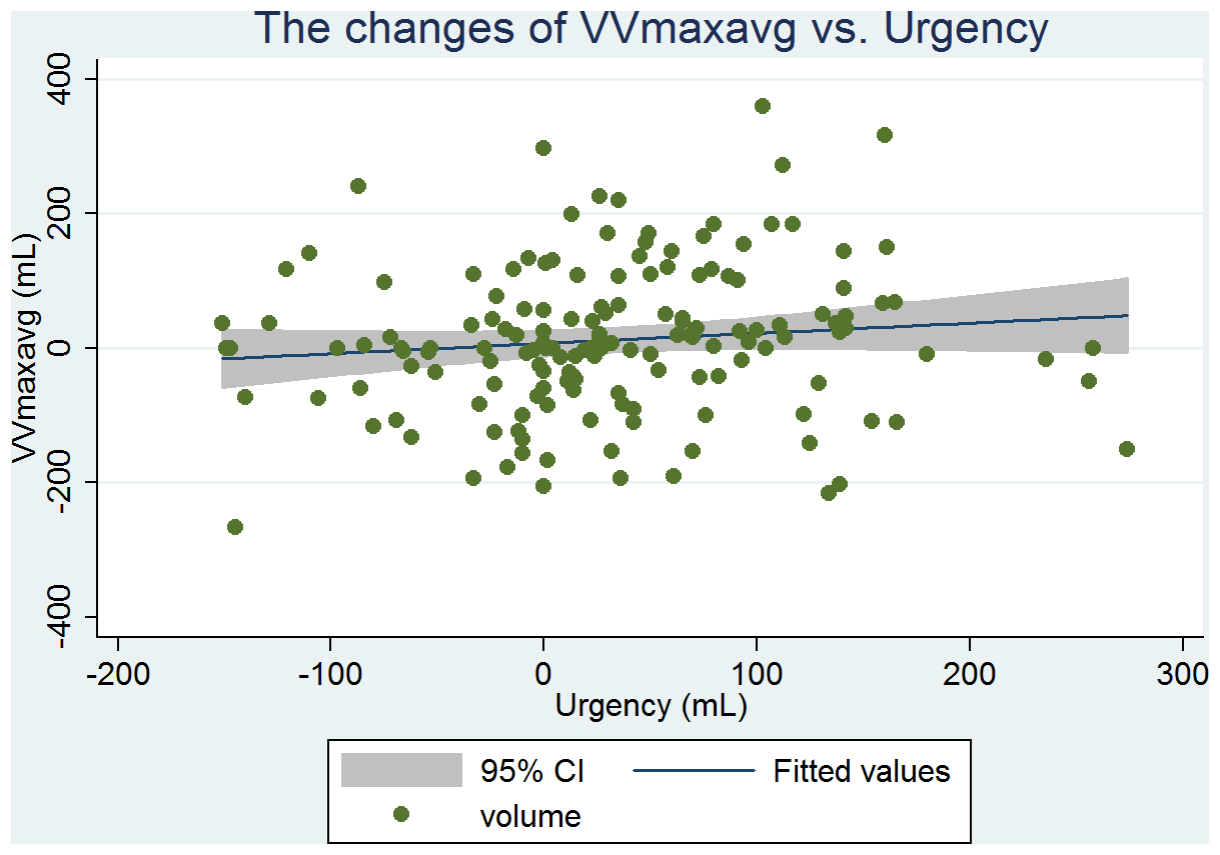
Regression fit scatter plot of the change from baseline in VVmaxavg vs. the change from baseline in urgency.

There were 158 patients that received follow-up assessments for monitoring therapeutic effects. The baseline and follow-up data for this group are shown in Table 3. Duration from baseline to follow-up was ≥12 weeks for all patients. Among this group, 75% (118/158) were diagnosed as having overactive bladder syndrome. The changes from baseline in DVVmaxavg were significantly correlated to that of VSD (ρ = 0.32, Figure 5). The changes from baseline in DVVmax for days 1 through 3 were also significantly correlated with that of VSD and had similar mean volumes (ρ = 0.22, 0.25, and 0.25, respectively). However, these significant correlations did not exist among the corresponding parameters of the 24-h (i.e., daytime and nighttime) bladder diary. In addition, the change from baseline in VVmaxavg was significantly correlated to that of urgency (mean 12 vs. 32 mL, ρ = 0.17, P = 0.04, Table 3, Figure 6).

**Table 3:** Baseline and follow-up data from urodynamic studies and bladder diaries and the correlations between the changes in VSD, urgency and the bladder diary parameters in 158 women.

	Therapeutic effect				Correlation with change of VSD		Correlation with change of Urgency	
Variables	Pre-treatment	Post-treatment	Change	*P* †	ρ	*P* ‡	ρ	*P* ‡
Age (years)	60.1±39.1	–	–	–	–	–	–	–
Parity	2.9±1.4	–	–	–	–	–	–	–
VSD (ml)	245±78	270±81	25±69	<0.001	–	–	–	–
Urgency (ml)	319±91	351±110	32±82	<0.001	–	–	–	–
Day 1 VVmax (ml)	325±132	345±138	19±135	0.15	0.11	0.15	0.18	0.02
Day 2 VVmax (ml)	336±140	346±141	9±142	0.77	0.09	0.26	0.14	0.08
Day 3 VVmax (ml)	345±152	349±132	4±160	0.40	0.09	0.28	0.07	0.40
The maximum of days1–3 VVmax (ml)	398±155	406±150	8±149	0.59	0.13	0.11	0.15	0.06
VVmaxavg (ml)	335±124	347±123	12±111	0.38	0.12	0.14	0.17	0.04
Day 1 DVVmax (ml)	267±121	287±112	20±130	0.005	0.22	0.005	0.30	<0.001
Day 2 DVVmax (ml)	259±116	286±104	26±121	0.007	0.24	0.002	0.25	0.001
Day 3 DVVmax (ml)	248±123	275±114	29±130	0.002	0.25	0.002	0.20	0.01
The maximum of days1–3 DVVmax (ml)	315±137	340±115	26±141	0.001	0.25	0.002	0.25	0.002
DVVmaxavg (ml)	258±102	283±93	22±99	<0.001	0.32	<0.001	0.32	<0.001

† Wilcoxon signed-rank test.

‡ The changes of voided volumes of bladder diaries were compared with the changes of the volume at strong desire to void or urgency by Spearman rank-correlation coefficient.

Abbreviations same as Table 1.

**Figure 7:**
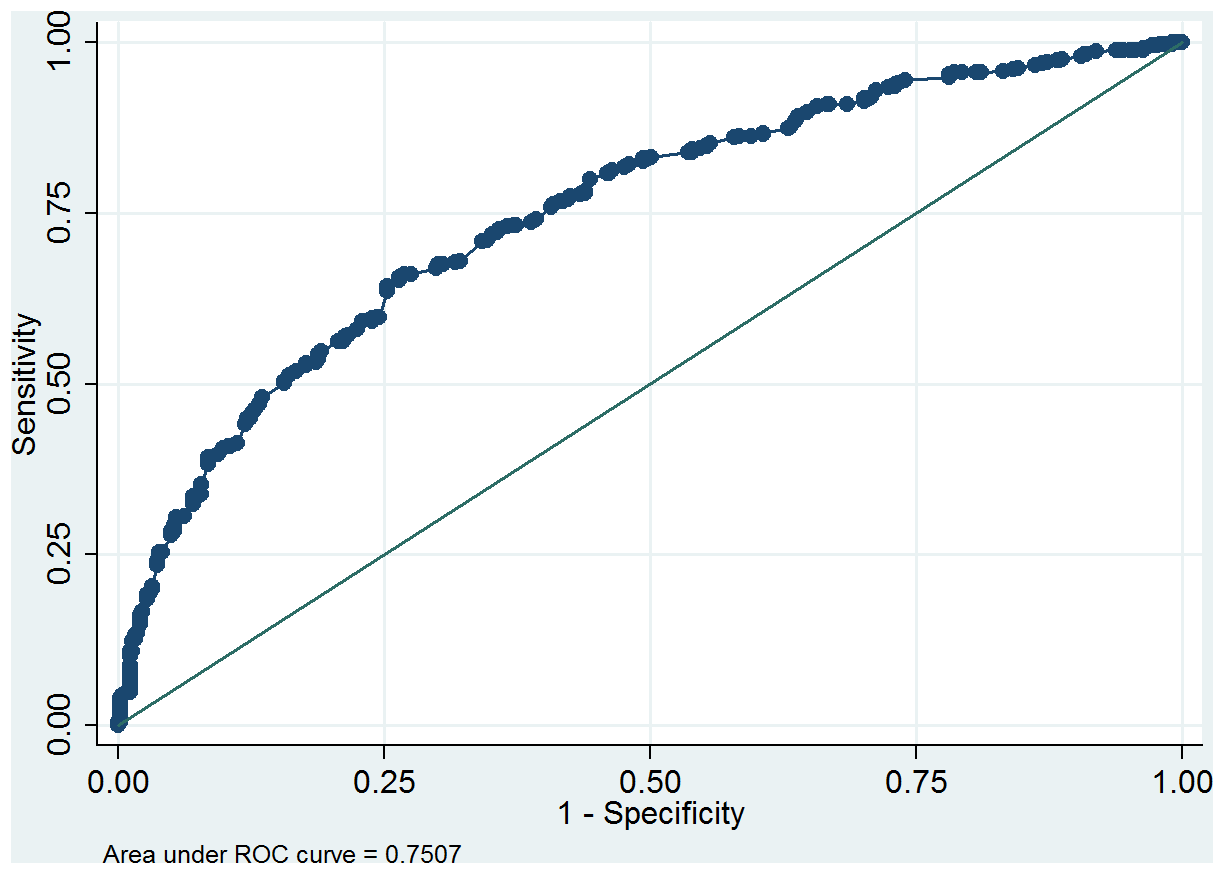
Receiver operating characteristic (ROC) curve for diagnosing bladder oversensitivity for the average of the daytime maximum voided volume excluding the first morning void.

**Figure 8:**
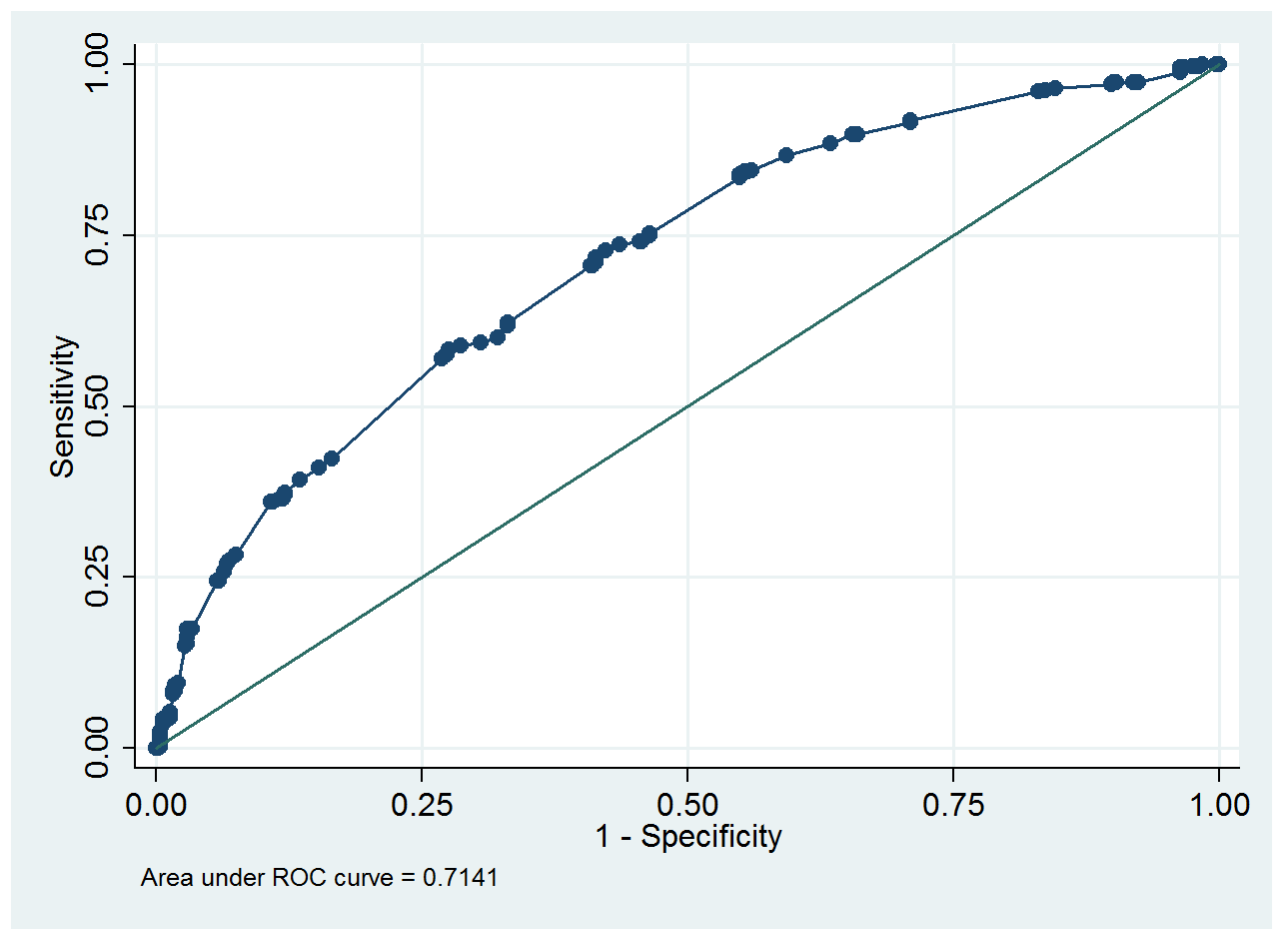
Receiver operating characteristic (ROC) curve for diagnosing bladder oversensitivity for day 1 daytime maximum voided volume excluding the first morning void.

DVVmaxavg had the highest receiver operating characteristic curve (ROC) area for predicting bladder oversensitivity (0.75, 95% confidence interval = 0.72–0.78, Figure 7). The DVVmaxavg cutoff point of 250 ml (sensitivity 70.9%, specificity 65.8%) had a good predictive value for detecting bladder oversensitivity. The ROC area of day 1 DVVmax was 0.71 (95% confidence interval = 0.68–0.75, Figure 8). The day 1 DVVmax cutoff point of 250 ml (sensitivity 70.6%, specificity 59.1%) also had a good predictive value for detecting bladder oversensitivity.

The correlation coefficients of the VVmax and DVVmax between day 1, day 2, day 3 and their averages were good with a range of 0.70 to 0.91 (all P<0.001, Table 4 and 5).

**Table 4:** Correlation matrix of day 1, day 2, day 3 VVmax and their averages (n = 900).

	Day 1 VVmax (ml)	Day 2 VVmax (ml)	Day 3 VVmax (ml)
Day 2 VVmax (ml)	0.72†	–	–
Day 3 VVmax (ml)	0.70†	0.75†	–
VVmaxavg (ml)	0.88†	0.91†	0.91†

† Spearman rank-correlation coefficient (ρ), and all *P* are <0.001.

Abbreviations same as Table 1.

**Table 5:** Correlation matrix of day 1, day 2, day 3 DVVmax and their averages (n = 900).

	Day 1 DVVmax (ml)	Day 2 DVVmax (ml)	Day 3 DVVmax (ml)
Day 2 DVVmax (ml)	0.70†	–	–
Day 3 DVVmax (ml)	0.65†	0.71†	–
DVVmaxavg (ml)	0.88†	0.89†	0.88†

† Spearman rank-correlation coefficient (ρ), and all *P* are <0.001.

Abbreviations same as Table 1.

## Discussion

Yoon and Swift reported a good correlation between maximum bladder capacities determined from bladder diary and filling cystometry (ρ = 0.47, *P*<0.001) [15]. However, in a similar study, Ertberg et al. reported the same correlation to be poor [4]. In our study, we found that the maximum daytime void volume excluding first morning void was well-correlated with VSD determined by filling cystometry (ρ = 0.43 to 0.51, all *P*<0.001), similar to Yoon and Swift [15]. Ertberg et al. and Yoon and Swift both reported that the bladder diary capacity was significantly higher than cystometry capacity (400 ml vs. 215 ml [median], *P* = 0.017 [4]; and 401 ml vs. 342 ml [mean] [15]). McCormack et al. reported that bladder capacity from cystoscopy was higher than that from bladder diary by 206 ml [16]. The significant differences between these different methods limit the ability to compare the bladder diary capacity data with the bladder capacity data determined by other methods. However, DVVmaxavg showed a similar mean volume and a linear correlation with VSD (Table 2, Figure 1), and therefore we can extrapolate VSD from the DVVmaxavg obtained from bladder diaries.

There have been a number of pharmacological studies using cystometry parameters to assess bladder capacity [5]–[9]. To our knowledge, there is a lack of evidence to suggest that a change from baseline in cystometric bladder capacity can be used to extrapolate bladder capacity determined from bladder diary [4]. In our study, the change from baseline in DVVmaxavg had a similar mean and was well-correlated with that of VSD (Table 3, Figure 5). Although the change from baseline in VVmaxavg was significantly correlated to that of urgency; however, the mean changes from baseline in DVVmaxavg and VSD were more similar (mean 22 vs. 25 mL, respectively), and their correlation efficient was higher (ρ = 0.32) than those of VVmaxavg and urgency (mean 12 vs. 32 mL, respectively; ρ = 0.17, Table 3). Thus, DVVmaxavg may be a better alternative parameter than VVmaxavg to evaluate the therapeutic outcomes of bladder storage disorders.

Mazurick et al. found no clinical benefit to extending a 1-day diary to 3 days [17]. van Melick et al. also reported that adding additional days to a 1-day diary did not add significant information [18]. Fitzgerald et al. reported that maximum voided volumes at subsequent days remained consistent with those from the first 24-h voiding diary [19]. In our study, the baseline DVVmax between day 1, day 2, day 3 and their average were well correlated (Table 5). In addition, the baseline and the change from baseline in day 1 DVVmax were all well correlated to those of DVVmaxavg (ρ = 0.88 and 0.74, respectively, all P<0.001). Therefore, our results agree with these earlier reports, and it may also be appropriate to use 1-day bladder diary to estimate DVVmax.

From the ROC curve analysis (Figure 7), DVVmaxavg <250 ml may be a good criterion to screen for bladder oversensitivity. Although DVVmaxavg had a higher ROC area, day 1 DVVmax <250 ml showed similar sensitivity and specificity and therefore also appears to be a good clinical alternative parameter for screening.

Our study was limited in that the study population was entirely Taiwanese, and therefore our results might not be able to be generalised to other populations. Also, the retrospective nature of this study may have biased the results. However, we believe the large sample size should make our results reliable. Third, cases with different life style could influence their voiding behaviour, such as toilet time control because of working or other reasons, different medicine administration, et al., and thus might bias the correlation between bladder dairy and cystometric parameters. Finally, the 3-day bladder diary may be difficult for some patients to complete correctively, especially those that have a job, and this may also have biased our findings.

### Conclusions

DVVmaxavg appears to be the bladder diary parameter best correlated with VSD. DVVmaxavg or day 1 DVVmax may be useful in screening for bladder oversensitivity and assessing efficacy of bladder storage disorder treatments.
